# Comparison of Individual Penalties According to Gender and Weight Categories of Elite Judo Athletes from Four World Championships

**DOI:** 10.3390/biology11091284

**Published:** 2022-08-29

**Authors:** Husnija Kajmovic, Damir Karpljuk, Safet Kapo, Jozef Simenko

**Affiliations:** 1 Faculty of Sport and Physical Education, University of Sarajevo, 71000 Sarajevo, Bosnia and Herzegovina; 2 Faculty of Sport, University of Ljubljana, 1000 Ljubljana, Slovenia; 3 School of Life and Medical Sciences, University of Hertfordshire, Hatfield AL10 9EU, UK

**Keywords:** combat sports, performance, competitions, negative judo, education, prevention

## Abstract

**Simple Summary:**

Penalties in judo (shido) have been previously associated with match outcomes and increased the likelihood of being defeated, particularly in heavier weight categories. Each 1-min increase in match duration and further athlete proceeds in competition increases the possibility of receiving a penalty. Penalties have also been associated with the occurrence of injuries, especially with grip fighting and other illegal moves and therefore, have a substantial effect on athletes’ health. The main findings highlighted that the leading penalties in all weight categories for both genders on Judo World Championships (WC) were Non-combativity, Avoid Grip and False Attack. Additionally, a new trend in heavyweight athletes with a lower number of penalties is noted.

**Abstract:**

Background: This research aimed to compare individual penalties by gender and weight categories in judo from the Judo World Championships (WC): Budapest—2017, Baku—2018, Tokyo—2019 and Budapest—2021 in all individual weight categories for females and males. Methods: Data were collected by notational analysis of 2041 penalty videos for females and 3473 penalty videos for males (total *n* = 5514). All individual penalties—Shido 1, 2, 3 and Hansoku Make (direct disqualification) were analysed by the Pearson chi-square test at the level of statistical significance of 5%. Results: Significant differences were noted in the assigned individual penalties between individual categories (*p* < 0.001) in both genders. The significant difference was contributed mainly by the weight category +78 kg with penalties Non-combativity (5.3) and Avoid Grip (−3.4) in females, while in males it impacted by the +100 kg weight category and the Non-combativity (4.2) and Avoid Grip (−4.0) penalties. For females, the most dominant individual penalties were Non-combativity (41.6%), Avoid Grip (16.2%) and False Attack (15.0%), and were Non-combativity (40.3%), Avoid Grip (19.5%) and False Attack (16.4%) for males. The largest number of penalties in females were in −52 kg (16.7%), −57 kg (15.9%) and +78 kg (15.2%) categories, while in males, they were −66 kg (17.2%), −73 kg (16.1%) and −90 kg (15.6%). Conclusions: The findings of this study highlight the leading penalties in all weight categories for both genders on WC to be Non-combativity, Avoid Grip and False Attack. Additionally, a new trend in heavyweight athletes with a lower number of penalties is noted. The obtained results indicate the need to pay more attention to working with competitors of all ages and genders on education to implement tactical variants, forms and means to use penalties to athletes’ advantage, especially after a possible rule change and to lower the occurrence of injuries.

## 1. Introduction

Judo presents a dynamic sport where victory can be achieved by judoka scoring points—Ippon or two Waza-ari’s [1], which are the result of successfully performing throwing or ground techniques (levers, chokes and immobilisation) [1]. Additionally, victory can be achieved by obtaining lighter (Shido) and severe (Hansoku-make—direct disqualification) penalties due to negative fighting, obstruction of the fight or violation of the spirit of judo [1].

The literature emphasises the existence of several intentions of judo rules, and they are: protection of competitors from injury, providing the same and fair opportunities to competitors during the fights to achieve Ippon, making judo a dynamic and audience-friendly sport providing a new and inventive way in order for judo to evolve and continue to grow [2]. In general, the rules determine the four types of relationships of participants in competition [3], and they are as follows: with other participants (competitors, judges, coaches and audience), with space, with equipment and with the way they should adapt to time. The relationship between competitors and judges in terms of penalties is especially interesting research and was the main focus of the present study. Therefore, penalties in judo are considered a critical tactical variable and one of the essential and very effective tactical skills and strategies that enables a judoka to enforce a penalty on the opponent and accumulate them to consequently win a match [4].

Receiving a penalty in judo (shido) is associated with match outcomes, increasing the likelihood of being defeated, particularly in heavier weight categories [5]. Additionally, each 1-min increase in match duration increases the possibility of receiving a shido [6]. This consequently means the longer the fight lasts and the further the athlete proceeds in the competition, there are more chances of receiving penalties. Penalties are also directly connected to 18.76% of all injuries reported in top-level judo competitions and therefore, have a substantial effect on athletes’ health [7], as it was reported that grip fighting produces 15.07% in addition to other illegal moves with 3.69% of injuries reported in top-level judo competitions [7].

The International Judo Federation (IJF) implemented six significant rule changes in judo from 2010 to 2020 [8]. These changes were intended to simplify judo for the public, as well as to devalue the use of penalties to achieve victory (2010 = koka’s exclusion; 2013 = penalty was no longer worth scores; 2017 = yuko’s exclusion, shido no longer decided the winner in regular time; 2018 = shido no longer decided the golden score winner) [8]. In general, IJF intent was to encourage positive judo [8]. However, if positive judo wants to be encouraged, negative actions must strictly be penalised. Studies at the beginning of this period analysing the 2013 European championship showed that the rule changes were not efficient as they increased penalties for both genders and decreased scoring [9]. Analysis of rule change between Grand Slam Paris 2016 and 2017 highlighted no significant differences in the penalties of “Hansoku-make” for men and women and a significant reduction in the total number of Shido penalties for men [10]. Another analysis between Olympic games (OG) London 2012 and Rio 2016 showed an increase in the number of penalties per athlete per match in both genders [11], while an analysis of world championships (WC) 2015 Astana and WC 2017 Budapest showed a decrease in the total number of penalties for women and men [12]. An analysis of weight categories from WC Astana to WC Budapest showed that extra-light weights (48 kg and −60 kg) received fewer penalties than lightweights upwards; heavyweights received more penalties than all other weight categories from middleweights downwards [12].

The reason for these penalties being awarded was analysed between the WC 2014 and WC 2015 for men, which showed contestants received the warning Shido 1 for Avoid Grip, Fighting One Handed, Hold Same Side; warning Shido 2 was awarded for Hold Same Side, False Attack, Defensive Posture and Avoid Grip; warning Shido 3 for False Attack, Hold Same Side, Defensive Posture and Avoid Grip [13]. Furthermore, the importance of penalties has been highlighted as penalties (shidos) are three times more frequent in the losers than in the winners [5], as receiving a shido during the match increased the possibility of losing [6] and having one or two penalties fighting standing up favours the opponent achieving a Waza-ari [14]. Additionally, it was highlighted that the importance of penalties increases the athlete’s progress in a competition [15].

It can be concluded that judo penalties have been the focus of judo research from several aspects and their usage in technical–tactical aspects is of great importance in top-level judo. However, detailed penalty overviews for all weight categories for males and females in world championships are scarce. Therefore, this study aims to compare individual penalties according to gender and weight categories in judo from the last four world championships.

## 2. Materials and Methods

### 2.1. Sample 

The sample of participants includes the total sum of penalties (*n* = 2041) with four world championships for female seniors, and by categories: −48 kg (*n* = 287), −52 kg (*n* = 340), −57 kg (*n* = 325), −63 kg (*n* = 296), −70 kg (*n* = 291), −78 kg (*n* = 192), +78 kg (*n* = 310); the total sum of penalties (*n* = 3473) with four world championships for male seniors, and by categories: −60 kg (*n* = 474), −66 kg (*n* = 596), −73 kg (*n* = 560), −81 kg (*n* = 535), −90 kg (*n* = 542), −100 kg (*n* = 425) and +100 kg (*n* = 341).

### 2.2. Method of Data Collection

The data were collected based on a notational analysis of videos of all individual penalties from the World Judo Championships held in 2017 in Budapest (Hungary), in 2018 in Baku (Azerbaijan), in 2019 in Tokyo (Japan) and in 2021 in Budapest (Hungary). Two international referees assessed each individual penalty by following the situation that forced the judge to award the penalty, the gesture of the judge with his hands when during this action, as well as the scoreboard on which the sentence was recorded. Cohen’s Kappa test results for estimating the agreement of two judges for women is 0.982, and for men is 0.984, representing a very good agreement between two judges in awarding penalties during the competition [16].

Penalties were recorded for all competitors (winners and losers) in the regular time of the match (4 min) and the golden score extension. They received penalties from the judges for committing minor or serious offences, thus violating the rules of judo defined by the International Judo Federation (IJF, 2017–2021). The data are published on the IJF official website for judo statistics (http://www.judobase.org; accessed on 10 May 2022). Because the data were provided from open access website using the public domain and athletes’ personal information was not reported, no ethical issues are present in analysing or interpreting these data since they were obtained in secondary form and not generated by experimentation [11,17,18,19]. Therefore, written informed consent was not needed. 

### 2.3. Sample Variables

The variables in this study are all individual penalties awarded by judges: Hold Trouser Leg, Hold Sleeve Ends, Avoid grip, Outside Contest Area, Defensive Posture, Escape With Head, Non-combativity, False Attack, Hold Same Side and Other Penalty: Pistol Grip Stretched Leg, Hand on Face, Disarrange Judogi, Holding Belt, Bear Hug, Fingers in Sleeve, Illegal Joint Lock, Leg Inside Blocking, Pull Down, Bend Opposite Fingers, Untidy Judogi, Illegal Newaza Entry, Push Out, Fingers Interlocked, Kick To Break Grip, Kicking, Metal Object, Unsportsmanlike conduct, Bridge and Head drive.

### 2.4. Data Analysis

Pearson’s chi-square test at the level of statistical significance of 5% was used to determine differences in individual penalties within the same genders and different weight categories. For analysing the strength of the association, Cramer’s V was implemented. In order to determine the significance of differences between cells in different weight categories, standardised residuals (Std. Residual) were calculated. Data were processed using SPSS 22.0 Premium (IBM Corporation, Armonk, NY, USA), and the tables show frequencies and percentage values.

## 3. Results

For female competitors, the most dominant individual penalties were Non-combativity (41.6%), Avoid Grip (16.2%) and False Attack (15.0%). For male competitors, the most dominant individual penalties were Non-combativity (40.3%), Avoid Grip (19.5%) and False Attack (16.4%). Additionally, the results in female competitors showed that the largest percentage of penalties in individual weight categories was in the −52 kg (16.7%), −57 kg (15.9%) and +78 kg (15.2%). Among male competitors, the largest percentage of penalties was awarded in the categories −66 kg (17.2%), −73 kg (16.1%) and −90 kg (15.6%).

Table 1 and Table 2 show the frequencies, percentages, and standardised residuals of individual penalties in all weight categories for seniors (men and women). The results showed the existence of statistically significant differences between all weight categories for female seniors in the awarded individual penalties (Pearson’s chi-square: 130.7; df: 54; *p* < 0.001; Cramer’s V = 0.103; *p* < 0.001), and also the existence of statistically significant differences between all weight categories for male seniors in the awarded individual penalties was determined (Pearson’s chi-square: 125.4; df: 54; *p* < 0.001; Cramer’s V = 0.078; *p* < 0.001).

By analysing individual cells in different weight categories, the standardised residuals showed that the statistically significant difference in female competitors was contributed mainly by the weight category +78 kg with the penalties Non-combativity (5.3) and Avoid Grip (−3.4). Male competitors standardised residuals showed the statistically significant difference is contributed mainly by the +100 kg weight category in the Non-combativity (4.2) and Avoid Grip (−4.0) categories.

## 4. Discussion

This research aimed to compare individual penalties according to gender and weight categories in judo from the World Judo Championships held in 2017, 2018, 2019 and 2021. The main results are that seniors differ within their weight categories in the penalties awarded. Of particular note are the Non-combativity penalties, which is the most convincingly awarded penalty, followed by the Avoid Grip, False Attack, Defensive Posture and Outside Contest Area penalties. Furthermore, the highest percentage of penalties in individual categories for females was achieved in the category −52 kg (16.7%), −57 kg (15.9%) and +78 kg (15.2%), and in the males, the highest percentage of penalties in individual categories was achieved in −66 kg (17.2%), −73 kg (16.1%) and −90 kg (15.6%). It is interesting that in heavier categories for women −78 kg (46.4%) and + 78 kg (61%) and men in −100 kg (45.4%) and +100 kg (54.8%), the percentage of Non-combativity penalties increases at the expense of reducing other penalties. The variable that contributes mostly to the difference between the weight categories is the Non-Combativity penalty and Avoid Grip in the +78 kg and +100 kg categories. The variable of Defensive Posture in the −81 kg category has been assigned many times and it contributes to the difference compared to other categories.

This phenomenon in larger weight categories was indicated by previous research [20] on Paris 2011 WC and showed that the largest number of penalties (45%) was in the heavyweight category, in the middleweight category (16.84%) and in the lightweight category (15.82%). However, our investigation shows that the highest number of penalties was awarded in the lightweight categories −66 kg in males (17.2%) and −52 kg in females (16.7%). This could indicate that heavyweight athletes started to rely more on scoring from technique than on imposing penalties on the opponent. Furthermore, an investigation of the number of penalties imposed during fights in men and women according to weight categories [21] found that the frequency of penalties for Non-combativity was significantly higher among men (65.4%) than in women (55.5%), which is in line with the current study results.

The current study results are also in line with results [22] from the OG Rio 2016, where in females, the most frequent penalties by categories are −48 kg -Non-combativity, Avoid Grip, False Attack; −52 kg: Non-combativity, Illegal Joint Lock, Defensive Posture; −57 kg: Avoid Grip, Outside Contest Area, False Attack; −63 kg: Non-combativity, False Attack, Illegal Joint Lock; −70 kg: Non-combativity, False Attack, Illegal Joint Lock; −78 kg: Non-combativity, False Attack, Defensive Posture; +78 kg: Non-combativity, Defensive Posture, Avoid Grip. In males [22], the most frequent penalties were: −60 kg: Avoid Grip, False Attack, Non-combativity; −66 kg: Non-combativity, False Attack, Avoid Grip; −73 kg: Non-combativity, Avoid Grip, False Attack; −81 kg: False Attack, Non-combativity, Defensive Posture; −90 kg: Non-combativity, Avoid Grip, Illegal Joint Lock; −100 kg: Non-combativity, False Attack, Outside Contest Area; +100 kg: Non-combativity, False Attack, Outside Contest Area. These results indicate that at these two highest levels of competition, OG and WC athletes rely on similar technical–tactical approaches as they result in similar most frequent penalties Non-Combativity, Avoid Grip, False Attack, Illegal Joint Lock and Outside Contest Area.

However, under the rules of fighting 2017–2020, the Illegal Joint Lock penalty was minimised and classified as other penalties. In this context, Stanković, et al. [23] investigated penalties of the World Top 10 ranked judoka from the IJF competition in 2018 in categories up to −90 kg. Results highlighted that for the elite athlete, the following penalties were the most frequently awarded: Passivity (49%), Bad Kumi-kata (20%), False attack (16%), Blocking attitude (6%), Stepping out of the contest area (5%) and other penalties (4%) which is in line with present study’s results. However, some differences in penalty categorisation need to be noticed; therefore, a direct comparison is impossible. Additionally, Callan, et al. [24] analysed penalties in lightweight female judo (−48 kg, −52 kg and −57 kg) from WC 2010 and WC 2014 and came to the indicator that the most dominant penalties were: Passivity, Avoid Grip, Defensive Posture, False Attack, Pull Down and Outside Contest Area. Compared to current research, it can be seen that the lightweight females’ category penalties of Non-combativity and Avoid Grip, were still the most frequent ones. However, penalties False attack is now more frequently awarded than Defensive Posture, which could be explained by the influence of new rules that promote more active judo.

In the study carried out by Escobar-Molina, et al. [5], male judo athletes exhibited a growing tendency to commit more penalties in heavier weight categories, especially for the last three categories. This trend has now changed as the present study results show that the +100 category had the lowest number of penalties awarded (9.8%) followed by the −100 kg (12.2%), with the rest of the categories showing constant frequencies. Female judo athletes previously showed a constant frequency of penalties across categories but with a greater number in the heaviest one [5]. However, the present study data shows that this ratio has slightly changed and is similar to the male category. The female heaviest category +78 kg dropped to the third place (15.2%), the −78 kg category on seventh place (9.4%) and the −70 kg category for sixth place (14.3%). This highlights that the judo rules have positively impacted the heavyweight categories by lowering the number of penalties. This confirms our statements about heavyweight athletes starting to rely more on scoring from technique than on imposing penalties on the opponent. This would also mean that previous findings and paradigms about heavyweight athletes must be reinvestigated. Furthermore, it was shown that a higher number of penalties compared to lightweight categories for the heaviest ones indicates that the combativeness decreases as the athletes’ weight category increases [25]. This was directly connected to the poorer physical fitness of heavyweight athletes [25] as they had lower performance in relative aerobic power, relative anaerobic power and capacity and relative maximal strength and muscle power compared to lightweight athletes [25,26]. Therefore, further research should investigate the new physiological profile of heavyweight athletes as the present study results indicate the change in activity which, according to previous research, indicated better physiological and technical performance.

The penalty structure in male competitors has changed compared to 2014–2015 WC [13], where Non-combativity, False Attack, and Outside Contest Area were the most frequent penalties compared to the present study findings with Non-combativity, Avoid Grip and False Attack. This indicates that more active judo with fewer penalties is taking place in the WC and confirms the findings from Balci and Ceylan [6]. It was also noted that in the −73 kg category of the 2017 WC, winners were prone not to be awarded a penalty (63.5%) while most of the defeated athletes were penalised with at least one Shido (51.4%) [14]. Authors have discussed that sometimes the athletes, when winning, learn how to avoid combat in the last minute of the fight by using illegal actions to prevent the opponent from scoring, but receiving penalties in this specific period [19]. This highlights the importance of active judo and how important it is to avoid “that first shido” in judo and to “save” penalties for the final minute or to go to the golden score with as low number of shido’s as possible. Similar findings were presented in the penalty ratios between the winner and non-winner athletes in WC, with the ratio of non-combativity and false attack significantly increasing for the second shido versus the First one, while the ratio of avoiding grip decreased [27]. It is necessary to put pressure on the opponent, i.e., be aggressive throughout the fight, to force mistakes in guard, movement, or fight for a better body position in relation to the opponent and force him/her to exit the fighting area. An opponent who is under this type of pressure receives a penalty (Shido 1) or more penalties (Shido 2, Shido 3 or Hansokumake—direct disqualification), and they are consequently forced to fight more openly, thus opening the possibility for a more aggressive competitor to act in Tachi Waza or Ne-Waza transition. A similar opinion is shared by Escobar-Molina et al. [5], who claim that combativeness is crucial in avoiding penalty points and influencing opponents to execute them and forcing opponents to commit penalties is an increasingly common tactic of fighting in modern judo.

Avoid Grip, avoiding the guard in the heaviest weight category competitors (male and female), is the least awarded penalty compared to other categories. This is most likely due to their motor skills, such as speed of hand movements, because they are slower to avoid or break the grip compared to lower weight categories. When fighting for the guard, they immediately execute it and do not avoid it. The Penalty Defensive position has the greatest implementation in which the passive competitor makes a block with his hands on the opponent’s body above the belt, which dominates him with his guard, but due to that blocking, he is not able to realise a certain throwing technique. From this passive position, the competitor does nothing or is unable to respond to that guard by imposing his guard. The penalty, False attack, is awarded because when the tori is trying to perform the throwing technique, he does not have a real guard-grip with his opponent, does not disturb the balance of the opponent at all with inadequate rotation of the body, uses the movement for defence against a stronger opponent or tries to break their grip. It has been argued that such indicators might be related to differences in psychological preparation rather than physical ones [28,29].

Receiving a penalty in judo (shido) has been previously associated with match outcomes as it increased the likelihood of being defeated, particularly in heavier weight categories [5]. Each 1-min increase in match duration increases the possibility of receiving a shido [6], consequently the longer a fight lasts and the further athletes proceed in the competition, there is a greater possibility of penalties. The current study showed that grip fighting in both males and females was the second most awarded penalty and literature has reported that grip fighting produces 15.07% of all injuries reported in top-level judo competitions [7]. Additionally, illegal moves that are also penalised contribute 3.69% of injuries reported in top-level judo competitions [7]. Altogether, penalties are directly connected to 18.76% of all injuries reported in top-level judo competitions and therefore, have a substantial effect on athletes’ health. The obtained results indicate the need to pay more attention in working with competitors of all ages and genders on the education of tactical variants, forms and means on how to avoid or use penalties for the competitor’s advantage in various weight categories. Furthermore, future research on the role of penalties in judo should focus more on younger age categories of cadets and juniors of both genders. Research should also investigate top elite athletes’ technical–tactical approaches on avoiding and/or using penalties for better practical application and teaching methods for youth athletes and to lower the occurrence of injuries.

Authors must acknowledge some limitations of this study. The main fact remains that penalties in judo were analysed separately without relation to throwing and ground floor techniques [30]. Therefore, a clear picture of how penalties are used in a technical–tactical sense is not clear, and future research should consider these factors as a whole, especially in the last minute of the fight or in the golden score. In addition, future research should explore coaches’ and athletes’ attitudes and strategies towards penalties and whether or how they prepare their athletes in training for certain situations they might find themselves in. Furthermore, the number of matches has not been controlled and further analysis should take that into consideration.

## 5. Conclusions

Penalties in judo are a vital part of leading the fight in the competition for women and men, which requires systematic and long-term work on tactical education. The results of this study showed that leading penalties in all weight categories for both women and men on WC were as follows: Non-combativity, Avoid Grip and False Attack. Highlighted indicators of the most common reasons why these particular penalties were awarded can help coaches and competitors recognise their weaknesses and turn them into strengths in terms of strength and conditioning training, technical performance quality, tactical actions during fights and to lower the occurrence of injuries. Additionally, a new trend in heavyweight athletes with a lower number of penalties is noted. Therefore, any change in the rules in judo must be accompanied by the tactical education of coaches and competitors of all ages and both genders through the training process, and in that way, the competitors would make a lower number of penalties, and thus the fights would be more interesting for the audience.

## Figures and Tables

**Table 1:** Frequencies, percentage values and standardised residuals of individual penalties in all weight categories for female competitors.

FEMALE COMPETITORS		Weight Category (kg)							Total
PENALTY		−48 kg	−52 kg	−57 kg	−63 kg	−70 kg	−78 kg	+78 kg	
Hold Trouser Leg	Count	7	7	5	6	6	4	2	37
	% within Penalty	18.90%	18.90%	13.50%	16.20%	16.20%	10.80%	5.40%	100.00%
	% within Category	2.40%	2.10%	1.50%	2.00%	2.10%	2.10%	0.60%	1.80%
	Std. Residual	0.8	0.3	−0.4	0.3	0.3	0.3	−1.5	
Hold Sleeve Ends	Count	11	12	6	14	3	3	1	50
	% within Penalty	22.00%	24.00%	12.00%	28.00%	6.00%	6.00%	2.00%	100.00%
	% within Category	3.80%	3.50%	1.80%	4.70%	1.00%	1.60%	0.30%	2.40%
	Std. Residual	1.5	1.3	−0.7	2.5	−1.5	−0.8	−2.4	
Avoid Grip	Count	59	64	62	38	56	25	26	330
	% within Penalty	17.90%	19.40%	18.80%	11.50%	17.00%	7.60%	7.90%	100.00%
	% within Category	20.60%	18.80%	19.10%	12.80%	19.20%	13.00%	8.40%	16.20%
	Std. Residual	1.8	1.2	1.3	−1.4	1.3	−1.1	−3.4	
Defensive Posture	Count	20	33	24	22	16	21	20	156
	% within Penalty	12.80%	21.20%	15.40%	14.10%	10.30%	13.50%	12.80%	100.00%
	% within Category	7.00%	9.70%	7.40%	7.40%	5.50%	10.90%	6.50%	7.60%
	Std. Residual	−0.4	1.4	−0.2	−0.1	−1.3	1.7	−0.8	
Escape With Head	Count	3	8	5	4	3	3	4	30
	% within Penalty	10.00%	26.70%	16.70%	13.30%	10.00%	10.00%	13.30%	100.00%
	% within Category	1.00%	2.40%	1.50%	1.40%	1.00%	1.60%	1.30%	1.50%
	Std. Residual	−0.6	1.3	0.1	−0.2	−0.6	0.1	−0.3	
Outside Contest Area	Count	18	14	27	23	30	13	14	139
	% within Penalty	12.90%	10.10%	19.40%	16.50%	21.60%	9.40%	10.10%	100.00%
	% within Category	6.30%	4.10%	8.30%	7.80%	10.30%	6.80%	4.50%	6.80%
	Std. Residual	−0.3	−1.9	1	0.6	2.3	0	−1.5	
False Attack	Count	36	66	59	37	45	22	41	306
	% within Penalty	11.80%	21.60%	19.30%	12.10%	14.70%	7.20%	13.40%	100.00%
	% within Category	12.50%	19.40%	18.20%	12.50%	15.50%	11.50%	13.20%	15.00%
	Std. Residual	−1.1	2.1	1.5	−1.1	0.2	−1.3	−0.8	
Non-Combativity	Count	109	113	114	129	107	89	189	850
	% within Penalty	12.80%	13.30%	13.40%	15.20%	12.60%	10.50%	22.20%	100.00%
	% within Category	38.00%	33.20%	35.10%	43.60%	36.80%	46.40%	61.00%	41.60%
	Std. Residual	−1	−2.4	−1.8	0.5	−1.3	1	5.3	
Hold Same Side	Count	14	8	11	9	10	5	4	61
	% within Penalty	23.00%	13.10%	18.00%	14.80%	16.40%	8.20%	6.60%	100.00%
	% within Category	4.90%	2.40%	3.40%	3.00%	3.40%	2.60%	1.30%	3.00%
	Std. Residual	1.9	−0.7	0.4	0.1	0.4	−0.3	−1.7	
Other Penalty	Count	10	15	12	14	15	7	9	82
	% within Penalty	12.20%	18.30%	14.60%	17.10%	18.30%	8.50%	11.00%	100.00%
	% within Category	3.50%	4.40%	3.70%	4.70%	5.20%	3.60%	2.90%	4.00%
	Std. Residual	−0.5	0.4	−0.3	0.6	1	−0.3	−1	
Total	Count	287	340	325	296	291	192	310	2041
	% within Penalty	14.10%	16.70%	15.90%	14.50%	14.30%	9.40%	15.20%	100.00%
	% within Category	100.00%	100.00%	100.00%	100.00%	100.00%	100.00%	100.00%	100.00%

**Table 2:** Frequencies, percentage values and standardised residuals of individual penalties in all weight categories for male competitors.

MALE COMPETITORS		Weight Category (kg)							Total
PENALTY		−60 kg	−66 kg	−73 kg	−81 kg	−90 kg	−100 kg	+100 kg	
Hold Trouser Leg	Count	13	13	16	18	10	1	10	81
	% within Penalty	16.00%	16.00%	19.80%	22.20%	12.30%	1.20%	12.30%	100.00%
	% within Category	2.70%	2.20%	2.90%	3.40%	1.80%	0.20%	2.90%	2.30%
	% of Total	0.40%	0.40%	0.50%	0.50%	0.30%	0.00%	0.30%	2.30%
	Std. Residual	0.6	−0.2	0.8	1.6	−0.7	−2.8	0.7	
Hold Sleeve Ends	Count	10	9	7	3	11	5	3	48
	% within Penalty	20.80%	18.80%	14.60%	6.30%	22.90%	10.40%	6.30%	100.00%
	% within Category	2.10%	1.50%	1.30%	0.60%	2.00%	1.20%	0.90%	1.40%
	% of Total	0.30%	0.30%	0.20%	0.10%	0.30%	0.10%	0.10%	1.40%
	Std. Residual	1.3	0.3	−0.3	−1.6	1.3	−0.4	−0.8	
Avoid Grip	Count	83	144	117	100	113	86	34	677
	% within Penalty	12.30%	21.30%	17.30%	14.80%	16.70%	12.70%	5.00%	100.00%
	% within Category	17.50%	24.20%	20.90%	18.70%	20.80%	20.20%	10.00%	19.50%
	% of Total	2.40%	4.10%	3.40%	2.90%	3.30%	2.50%	1.00%	19.50%
	Std. Residual	−1	2.6	0.8	−0.4	0.7	0.3	−4	
Defensive Posture	Count	24	35	31	43	21	20	23	197
	% within Penalty	12.20%	17.80%	15.70%	21.80%	10.70%	10.20%	11.70%	100.00%
	% within Category	5.10%	5.90%	5.50%	8.00%	3.90%	4.70%	6.70%	5.70%
	% of Total	0.70%	1.00%	0.90%	1.20%	0.60%	0.60%	0.70%	5.70%
	Std. Residual	−0.6	0.2	−0.1	2.3	−1.8	−0.8	0.8	
Escape With Head	Count	5	5	7	7	13	4	6	47
	% within Penalty	10.60%	10.60%	14.90%	14.90%	27.70%	8.50%	12.80%	100.00%
	% within Category	1.10%	0.80%	1.30%	1.30%	2.40%	0.90%	1.80%	1.40%
	% of Total	0.10%	0.10%	0.20%	0.20%	0.40%	0.10%	0.20%	1.40%
	Std. Residual	−0.6	−1.1	−0.2	−0.1	2.1	−0.7	0.6	
Outside Contest Area	Count	19	40	35	27	44	24	21	210
	% within Penalty	9.00%	19.00%	16.70%	12.90%	21.00%	11.40%	10.00%	100.00%
	% within Category	4.00%	6.70%	6.30%	5.00%	8.10%	5.60%	6.20%	6.00%
	% of Total	0.50%	1.20%	1.00%	0.80%	1.30%	0.70%	0.60%	6.00%
	Std. Residual	−1.8	0.7	0.2	−0.9	2	−0.3	0.1	
False Attack	Count	87	104	100	95	81	66	36	569
	% within Penalty	15.30%	18.30%	17.60%	16.70%	14.20%	11.60%	6.30%	100.00%
	% within Category	18.40%	17.40%	17.90%	17.80%	14.90%	15.50%	10.60%	16.40%
	% of Total	2.50%	3.00%	2.90%	2.70%	2.30%	1.90%	1.00%	16.40%
	Std. Residual	1.1	0.6	0.9	0.8	−0.8	−0.4	−2.7	
Non-Combativity	Count	190	196	214	216	205	193	187	1401
	% within Penalty	13.60%	14.00%	15.30%	15.40%	14.60%	13.80%	13.30%	100.00%
	% within Category	40.10%	32.90%	38.20%	40.40%	37.80%	45.40%	54.80%	40.30%
	% of Total	5.50%	5.60%	6.20%	6.20%	5.90%	5.60%	5.40%	40.30%
	Std. Residual	−0.1	−2.9	−0.8	0	−0.9	1.6	4.2	
Hold Same Side	Count	13	18	8	11	17	8	5	80
	% within Penalty	16.30%	22.50%	10.00%	13.80%	21.30%	10.00%	6.30%	100.00%
	% within Category	2.70%	3.00%	1.40%	2.10%	3.10%	1.90%	1.50%	2.30%
	% of Total	0.40%	0.50%	0.20%	0.30%	0.50%	0.20%	0.10%	2.30%
	Std. Residual	0.6	1.2	−1.4	−0.4	1.3	−0.6	−1	
Other Penalty	Count	30	32	25	15	27	18	16	163
	% within Penalty	18.40%	19.60%	15.30%	9.20%	16.60%	11.00%	9.80%	100.00%
	% within Category	6.30%	5.40%	4.50%	2.80%	5.00%	4.20%	4.70%	4.70%
	% of Total	0.90%	0.90%	0.70%	0.40%	0.80%	0.50%	0.50%	4.70%
	Std. Residual	1.6	0.8	−0.3	−2	0.3	−0.4	0	
Total	Count	474	596	560	535	542	425	341	3473
	% within Penalty	13.60%	17.20%	16.10%	15.40%	15.60%	12.20%	9.80%	100.00%
	% within Category	100.00%	100.00%	100.00%	100.00%	100.00%	100.00%	100.00%	100.00%
	% of Total	13.60%	17.20%	16.10%	15.40%	15.60%	12.20%	9.80%	100.00%

## Data Availability

The raw data supporting the conclusions of this article will be made available by the authors, without undue reservation, to any qualified researcher.
